# A cluster-randomised, controlled trial to assess the impact of a workplace osteoporosis prevention intervention on the dietary and physical activity behaviours of working women: study protocol

**DOI:** 10.1186/1471-2458-13-405

**Published:** 2013-04-29

**Authors:** Ai May Tan, Anthony D LaMontagne, Rani Sarmugam, Peter Howard

**Affiliations:** 1 McCaughey VicHealth Centre for Community Wellbeing, Melbourne School of Population and Global Health, University of Melbourne, Melbourne, VIC, 3010, Australia; 2 Health Promotion Board, Singapore, 168937, Singapore; 3 Melbourne School of Population and Global Health, University of Melbourne, Melbourne, VIC, 3010, Australia

**Keywords:** Osteoporosis prevention, Cluster randomised trial, Premenopausal women, Workplace, Calcium intake, Physical activity

## Abstract

**Background:**

Osteoporosis is a debilitating disease and its risk can be reduced through adequate calcium consumption and physical activity. This protocol paper describes a workplace-based intervention targeting behaviour change in premenopausal women working in sedentary occupations.

**Method/Design:**

A cluster-randomised design was used, comparing the efficacy of a tailored intervention to standard care. Workplaces were the clusters and units of randomisation and intervention. Sample size calculations incorporated the cluster design. Final number of clusters was determined to be 16, based on a cluster size of 20 and calcium intake parameters (effect size 250 mg, ICC 0.5 and standard deviation 290 mg) as it required the highest number of clusters.

Sixteen workplaces were recruited from a pool of 97 workplaces and randomly assigned to intervention and control arms (eight in each). Women meeting specified inclusion criteria were then recruited to participate. Workplaces in the intervention arm received three participatory workshops and organisation wide educational activities. Workplaces in the control/standard care arm received print resources. Intervention workshops were guided by self-efficacy theory and included participatory activities such as goal setting, problem solving, local food sampling, exercise trials, group discussion and behaviour feedback.

Outcomes measures were calcium intake (milligrams/day) and physical activity level (duration: minutes/week), measured at baseline, four weeks and six months post intervention.

**Discussion:**

This study addresses the current lack of evidence for behaviour change interventions focussing on osteoporosis prevention. It addresses missed opportunities of using workplaces as a platform to target high-risk individuals with sedentary occupations. The intervention was designed to modify behaviour levels to bring about risk reduction. It is the first to address dietary and physical activity components each with unique intervention strategies in the context of osteoporosis prevention. The intervention used locally relevant behavioural strategies previously shown to support good outcomes in other countries. The combination of these elements have not been incorporated in similar studies in the past, supporting the study hypothesis that the intervention will be more efficacious than standard practice in osteoporosis prevention through improvements in calcium intake and physical activity.

## Background

Osteoporosis is a disease characterised by bone fragility due to low bone mass and a break down in the skeletal framework. It is a major public health problem affecting millions of people worldwide, with significant physical, psychosocial and financial consequences for the patient and the health care system [1]. Women are at higher risk of getting osteoporosis due to attainment of lower peak bone mass early in life and hormonal changes that occur at the menopause [1,2].

While osteoporosis is a disease with a strong genetic predisposition, calcium intake and physical activity are well-established modifiable risk factors operating through the maintenance of bone mass and skeletal integrity [1,2]. Evidence suggests that physical activity and calcium intake can affect not just bone mineral density, but also risk of osteoporotic fractures [3,4]. Prospective longitudinal studies have estimated that 23% of osteoporosis is attributable to physical inactivity [3] and that almost 10% of osteoporotic fractures are attributable to low dietary calcium intake [4]. This demonstrates that there are substantial preventable fractions on the order of 10% - 20% for osteoporosis and osteoporotic fractures, and that efforts to develop intervention strategies to achieve this are warranted, thus prompting recommendations for population-based interventions to promote adequate calcium intake and physical activity to prevent osteoporosis.

### Limitations in current evidence base

The majority of health promotion studies addressing osteoporosis prevention suffer from weak intervention designs and lack of methodological rigour. Many intervention strategies did not appear to be guided by behaviour change theory. None appear to have referenced past evidence to determine the level of behaviour change that is required to make an impact on the disease and its consequences [5-8]. Some interventions consisted of one-off information sessions or print resource distribution [5,9].

i) Single behaviour versus multiple behaviour approaches

None of the studies targeting osteoporosis prevention behaviours have attempted to approach the dietary and physical activity components separately. They adopted the same intervention strategies for both behaviours and did not appear to have incorporated unique strategies for either behaviour into their intervention design. These interventions reported modest or no increases in calcium intake in the short-term [5-7,10] and generally poor physical activity outcomes [5,10-12].

Evidence suggests that single health behaviour interventions were more effective at improving the targeted behaviours than multiple behaviour interventions [13]. Interventions that have singularly targeted dietary calcium intake for women have consistently reported positive outcomes [14,15]. Positive outcomes are also often reported in intervention studies specifically targeting general physical activity [16,17]. Few physical activity behaviour interventions have been carried out in the context of bone health in adult populations. Prescriptive exercise interventions for adults, which included load bearing activity of moderate to vigorous intensity, have reported strong positive associations with improved bone mass [18]. However, prescriptive exercise interventions only engage participants in regimented exercise and do not address participants’ attitudes or barriers towards adopting physical activity. Such interventions consistently suffer high attrition rates and are not suited for implementation at the population level.

ii) Cognitive versus behavioural strategies

A meta-analysis of physical activity interventions suggests that behavioural strategies (such as goal setting, problem solving) are superior to cognitive strategies [19]. Taken together, these studies suggest that an osteoporosis prevention intervention design should place specific emphasis on behavioural strategies targeting calcium intake and physical activity as unique and distinct health behaviours.

iii) Occupational settings

Workplaces are valuable settings for the efficient delivery of preventive health intervention programs to healthy adult populations. Women in sedentary occupations are a priority group for osteoporosis prevention, as being both female and sedentary are independent risk factors for low bone mass and osteoporosis. Occupational sitting has been associated with low bone mineral density of the hip [20]. Workplaces with predominantly sedentary employees present great opportunities to address behaviours that can decrease the risk of osteoporosis. There are no published studies to date on workplace-based osteoporosis prevention programs. While most studies targeted women in the community, none of them targeted those with sedentary occupations.

iv) Osteoporosis prevention studies in Singapore

Research resources on osteoporosis prevention in Singapore were predominantly allocated to bio-medical interventions at the time of this Singapore-based study. Relevant health promotion studies in Singapore were limited and predominantly investigated knowledge and attitudes. No local studies had previously investigated the efficacy of behaviour strategies for osteoporosis prevention.

### Importance of this study for osteoporosis prevention at population level

Existing evidence points to unrealised potential in both intervention design and settings when addressing osteoporosis prevention. This study improves on previous research as follows:

1. It is the first to address dietary and physical activity components each with unique intervention strategies in the context of osteoporosis prevention.

2. The intervention design for both behaviours is guided by Bandura's self-efficacy theory. The design focused on behavioural strategies rather than cognitive strategies to increase subjects' self-efficacy to change behaviour.

3. The utilisation of a workplace platform to address the risk associated with low levels of physical activity at work.

4. The use of an evidence-based approach when setting the intervention outcomes. Targeted behavioural outcomes were supported by evidence with the potential to affect the burden of osteoporosis.

5. This study compared a strengthened intervention design to a standard care control, which was current conservative practice. The results would indicate the degree to which the intervention design improves on current practice.

### Aims

The overall aim of this study was to determine the efficacy of a tailored and self-efficacy focussed workplace-based intervention compared with standard care (print resources) in increasing the calcium intake and physical activity level of women with sedentary occupations.

The specific objectives were:

1. To test the hypothesis that a tailored workplace based intervention incorporating specific behavioural strategies for calcium intake and physical activity is more efficacious than standard care (simple print resource distribution) in increasing the calcium intake and physical activity levels.

2. To explore the relationship between self-efficacy scores for calcium intake and physical activity with actual calcium intake and physical activity levels to determine the extent to which self-efficacy mediates intervention-associated changes in calcium intake and physical activity.

### Study design

This was a prospective two-arm cluster randomised trial. Clusters were workplaces that were randomly assigned to receive either i) tailored workplace-based intervention or ii) print resources (standard care control arm).

### Specification of intervention targets

i) Calcium Intake

In 2004, the Singapore National Nutrition Survey reported the mean daily calcium intake of the female population as 598 milligrams [21]. This level of calcium consumption was below the recommended daily allowance (RDA) of 800 milligrams for 25 to 44 year old women who constituted the main target group for the study. The survey reported that 55.9% of the Singapore female population did not achieve sufficient calcium intake (defined as <70% of RDA) through their diet [21]. Fifty percent of women in the premenopausal age group (30 to 49 years old) had daily intake of 560.5 mg or less (range 258 mg to 565 mg) [21]. We anticipated that the women in our study were similar, and that a deficit of at least 250 mg needs to be corrected. This assumption would be tested through assessment of baseline calcium intake from both intervention groups.

Evidence supports the health significance of this study’s proposed effect size for calcium intake. Warensjo et al. (2011) reported on a 19 year follow up on 61,433 Sweden women and found that almost 10% of hip fracture may be attributable to low calcium intake (first quintile) [4]. It is important to note that the first quintile in this study was reported to be less than 759 mg per day [4]. This is high compared to Singapore female population where the mean intake in the first quartile is 411 mg per day [21]. Population attributable risk is potentially higher in the Singaporean female population due to lower calcium intake (e.g. because of lower dairy intake compared to Sweden). According to Warensjo et al. (2011), population attributable risk (%) of hip fracture decreased by 3.34% with every 300 mg increase in calcium intake [4].

This is further supported by studies in another population with calcium intake comparable to Singapore. Rouzi et al. (2012) studied independent predictors of all osteoporosis-related fractures among 707 healthy Saudi postmenopausal women over 5.5 years. They reported a mean daily calcium intake of 532 milligrams in their study population; very similar to the 598 milligrams reported in Singapore's female population [22]. The study estimated that 26.4% of osteoporotic fractures are independently attributable to low dietary calcium intake (<391 mg/day) [23]. The reported osteoporotic fracture relative risk (RR) is 1.66 for a low dietary calcium intake (<=391 mg/day) when compared to a higher intake (>/=648 mg/day) [23].

In Hong Kong, Lau et al. (1988) reported hip fracture RR to be 1.9 when comparing calcium intake in the lowest quintile (<75 mg/day) to the highest quintile (>244 mg/day) [24]. Chan et al. (1998) reported the odds of vertebra fracture to double (OR = 2.1) when dietary calcium intake was in the lowest quartile (<249 mg/day) compared to the highest quartile (>382 mg/day) [25].

Calcium consumption in the Asian population appears to be lower than in Europe [4,23-25], supporting a particular need for intervention in low calcium intake (e.g., many Asian) populations. Evidence has demonstrated that even a modest increase in calcium intake (in the range of 120-150 mg) can have substantial impact on osteoporotic fracture risk [23-25].

Past interventions have demonstrated that the effect size of 250 mg is achievable. Osteoporosis prevention studies that focused only on dietary interventions and incorporated strong behavioural strategies reported significant increases in calcium intake (200-300 mg) compared to controls [14,15].

ii) Physical activity

The Singapore National Health Survey in 2004 reported that 54.5% of the female population does not participate in leisure physical activity [22]. Only 18.8% of women in the pre-menopausal age group (30 to 49 years old) reported at least 60 minutes of physical activity per week [22]. The World Health Organisation (WHO) recommends that adults aged 18–64 should do at least 150 minutes of moderate-intensity aerobic physical activity throughout the week or do at least 75 minutes of vigorous-intensity aerobic physical activity throughout the week [26]. This means that majority of Singapore's female physical activity level falls well below the recommended guidelines. We anticipated that the women in our study sample would have a similar physical activity profile.

This study's intervention content was aimed at supporting participants to achieve a 60-minute increase in load-bearing physical activity of moderate to vigorous intensity. Evidence suggests that the risk of hip fracture declines 6% for every increase of 3 MET hr/week, which is equivalent to 60 minutes per week walking at an average pace [27].

A larger effect size was initially considered. However, participants in this study were anticipated to have very low physical activity at baseline. WHO recommendations state that inactive people should start with small amounts of physical activity and gradually increase duration, frequency and intensity over time [26]. Moreover, this study targets a domain of physical activity that is more site-specific and higher in intensity, hence potentially more challenging to adopt.

Large worksite interventions to increase general physical activity have reported a wide variation in improvements. Reported increases in moderate to vigorous intensity physical activity per week range from 40 to 300 minutes [28,29]. Differences in intervention design and duration might account for this variability. Notably, none of these workplace based studies were designed for osteoporosis prevention. None of the studies investigating osteoporosis prevention behaviours reported physical activity outcomes in duration or intensity.

In summary, the effect size of 60 minutes of moderate to vigorous intensity load -bearing physical activity is achievable for a workplace-based intervention, and has the potential to meaningfully reduce the risk of osteoporosis.

### Sample size calculation

Sample size calculations took into consideration the cluster randomisation design by incorporating the design effect into the calculation. The design effect was calculated based on a cluster size of 20 and the intracluster correlation coefficient (ICC) using the formula: Design Effect = 1 + (within cluster sample size −1) x ICC. In the absence of ICCs for the outcome measures, available population standard deviations were used to calculate the variances and the ICC using the formula: ICC = variance between cluster/(variance between cluster + variance within cluster). In this study, there were two primary outcome measures, calcium intake and physical activity. Sample size calculations were carried out for both measures. Calculations were based on α = 0.05 and β = 0.1. Table 1 shows the parameters used for calculating the number of clusters and the total number of participants. The study deliberately planned to over recruit within clusters to factor in a 30% attrition rate.

**Table 1:** Summary of parameters used in the sample size calculation

**Outcome measures and mediators**	**Effect size**	**ICC used in sample size calculation**	**Standard deviation used in sample size calculation**	**Design effect**	**Number of clusters**	**Total subjects to be recruited**	
						**Before factoring in 30%attrition**	**After factoring in 30%attrition**
Calcium intake (milligrams per day)	250 milligrams	0.5*	290 milligrams***	10.5	14.7	294	480
Physical activity duration (minutes per week)	60 minutes	0.05**	150 minutes****	1.95	13	255	420

*Calculated using standard deviation from National Nutrition Survey 1998 and based on conservative estimates of inter- and intra-cluster variance [23].

**Based on estimates from published studies [30,31].

***Estimate from National Nutrition Survey 1998 [32].

****Based on estimates from published studies [33,34].

The number of clusters required was different for each outcome measure, so the study was based on the highest number of clusters required, which was that for calcium intake (14.7). This was rounded up to 16 to ensure equal cluster numbers in both arms of this study.

There was an error in the final step of this study's sample size calculations. The calculation did not double the sample size calculation that yielded the number of required subjects per arm (for this two-arm study). The sample size calculation, however, was very conservative with the ICC estimates and the study was expected to be overpowered despite the omission of this step. This study still has 90% power to detect 355 milligrams increase in calcium intake and 85 minutes increase in moderate to vigorous intensity physical activity with 16 clusters of 20 participants per cluster.

### Eligibility of workplaces for entering the study

#### Workplace (cluster) inclusion criteria

•Workplaces in industry that was primarily office-based and sedentary in nature, such as government administration departments, publishing industry, property development and finance industry.

•Workplaces that were able to recruit at least 30 female employees engaged in desk-based jobs (sitting for at least 50% of working hours).

•Agreement to permit up to 10 hours of paid work time during the course of the study (12 months) for the recruited employees to participate in pre-post data collection and intervention activities.

### Eligibility for within-cluster recruitment

Within-cluster inclusion criteria were:

•Being female

•Age 25–49 years of age

•Being in a sedentary job (at least 50% of work hours seated)

Within-cluster exclusion criteria were:

•Being pregnant or lactating

•Diagnosed osteoporosis

•Diagnosed kidney problems

•Participation in another health program that addresses diet and/or physical activity

### Recruitment

#### Cluster (Workplace) recruitment

Clusters were sampled from database of workplaces who were recipients of 2003 Singapore Health Award. These workplaces would have demonstrated commitment to promoting employee health to receive this national award, hence the characterisation of this trial as assessing efficacy rather than effectiveness.

Generic invitation letters were mailed to 97 workplaces to invite them to participate in this study. The letter stated the objectives of the study but did not detail the nature of interventions. It stated that a briefing would be conducted to provide them with the details. A faxed reply from the workplace was requested to confirm participation by a stipulated date.

Thirty-seven faxed replies were received by the stipulated deadline. The workplaces’ names were arranged in a data sheet according to the date and time of receipt. Eligibility was on a first-come-first served basis. When sufficient clusters (workplaces) had been recruited, the subsequent workplaces who responded were placed on a reserve list also in order of the date and time of receipt. The workplaces (clusters) that have been recruited were labelled WP1 to WP16 according the date and time of the fax.

#### Cluster randomisation process

When the cluster recruitment process was completed, a statistician generated a set of random numbers for the list of workplaces recruited. The statistician had no access to the faxed replies and was blinded to the identities of the workplaces. The random numbers were generated for the labels WP1 to WP16. When the random number had been assigned to the workplace, the names of the workplaces were re-arranged according to the random numbers assigned in ascending order (smallest number first and biggest number last). The first eight workplaces in this new arrangement were assigned to the intervention group and the subsequent eight workplaces were assigned to the control group. Workplaces recruited were in government administration, property development, finance, publishing and energy provision industries.

**Figure 1:**
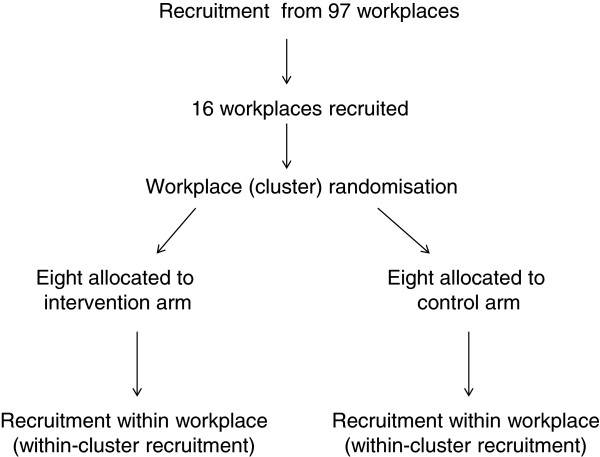
Recruitment and randomisation flow chart.

Following randomisation, workplace coordinators from the two groups were invited to two different briefings, depending on assignment to the intervention or control group. The briefings provided intervention and control group-specific information to respective workplace coordinators. The briefings also detailed the commitment required of the workplaces, the (within-workplace) recruitment process and the nature of assessments (data collection procedures). The workplaces in both groups were blinded to the type of intervention that the other groups were to receive. It was considered highly unlikely that unblinding would occur, as the workplaces were not geographically close. Figure 1 summarises the recruitment and randomisation process.

#### Within cluster recruitment (individuals)

When the workplace (cluster) allocation process was completed, workplace coordinators from both groups were contacted to commence the within-cluster recruitment process. The coordinator was provided with resources (such as posters, e-mailers) to publicise the recruitment. Employees who expressed interest were screened for eligibility before being recruited into the study. Over-recruitment by 30 percent to account for attrition was planned. Workplaces that recruited more than 30 respondents would create a waiting list in the event of withdrawal before commencement. Workplaces with less than 30 respondents would include all eligible respondents in the study.

### Ethics approval

The study was carried out as part of the Health Promotion Board Osteoporosis Prevention Programme initiative. The study was reviewed and approved by the Health Promotion Board (Singapore) Research and Ethics Committee before commencement.

### Consent

All recruited employees were provided with a consent form, accompanied by print information about the study. The content of the information sheet and their right to withdraw were explained to the recruits during their individual appointment with the investigator. Signed consent forms had to be returned to the investigator before the employees could formally enrol. The information sheet stated that the right of any subject to cease participation without giving reasons would be respected.

### Ethical considerations for the control group

Educational resource distribution was a strategy that was already in place for promoting bone health awareness in many Singaporean workplaces. Resources on osteoporosis prevention were widely distributed through various platforms such as community events, workplaces and health facilities. It would not be appropriate for workplaces in the control arm to receive less than an existing intervention, hence the standard care control.

The purpose of the study was to investigate if a more targeted and structured intervention with organisational support is more efficacious than existing practice. It was important to maintain the existing practices/strategy for the control group for the comparison to be purposeful, and to determine the extent to which current practice can be improved upon. These were the key ethical considerations for the study design.

### Data collection

Data for the two outcome measures, calcium intake and physical activity, were collected at three time points for both study arms. The mediators in this study, self-efficacy scores for calcium intake and physical activity, were also collected at the same time points. Demographic and lifestyle information were collected at baseline.

The three data collection points were:

•Baseline: four weeks before the intervention

•Four weeks after the intervention workshops (for the intervention group) were completed

•Six months after the first post-intervention data collection was completed

**Figure 2:**
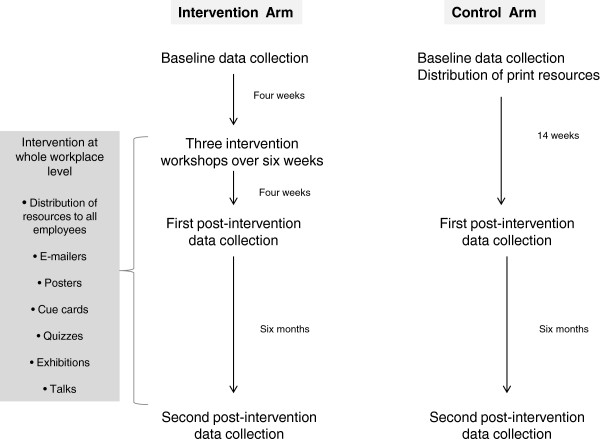
Data collection and intervention timeline.

Data collection for both the intervention and control arms took place during similar periods at every time point. Figure 2 provides an overview of the timeline for data collection.

### Outcomes measures

The outcome measures in this study are

•Daily dietary calcium intake (milligrams per day)

•Total moderate to vigorous load-bearing leisure time physical activity duration per week (minutes)

### Mediators

**Figure 3:**
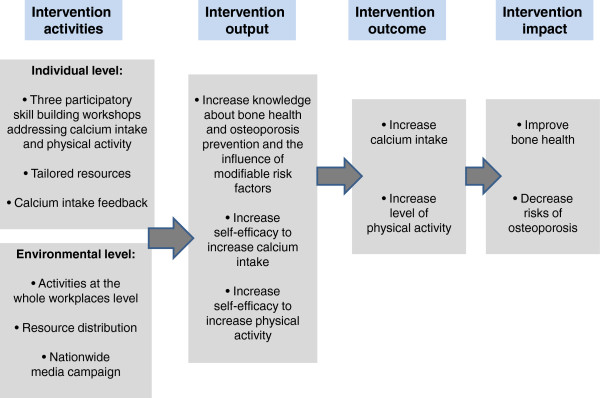
Logic model for the intervention group.

The hypothesised intervention-specific mediators of improvement in outcomes in this study are (see also Figure 3):

•Self-efficacy scores for calcium intake

•Self efficacy scores for exercise

### Dietary calcium intake data collection method

Calcium intake was measured using a three-day diet record. This method involved each participant keeping a detailed written record of the foods and beverages consumed over three days. Three day recording was selected as recording periods of more than three or four days were reported to be unreliable due to respondent fatigue [35]. Specific emphasis was put on the correct description of portion sizes so that an accurate estimate of calcium content could be derived.

An appointment was scheduled to meet each participant individually to provide specific instructions for completion of the three-day diet record. The three days would include two representative weekdays and one representative weekend day. Completed dietary records were collected and sent to a qualified nutritionist for analysis to establish the calcium content. The nutritionist was blinded to the treatment arms and the identities of the workplaces and participants.

### Physical activity duration data collection methods

Physical activity was measured using the EPIC Norfolk Physical Activity Questionnaire 2 (EPAQ-2). The EPAQ-2 was designed to measure the different sub-dimensions of physical activity in the Norfolk cohort of the European Prospective Investigation into Cancer (EPIC-Norfolk) in 1999 [36]. It is a self-reported questionnaire on disaggregated physical activity enabling the data be re-aggregated to the dimension of physical activity of interest, for example load-bearing activity of relevance to osteoporosis prevention. The EPAQ-2 has been validated against four-day heart rate measurement and was concluded to have the validity and repeatability/reliability to be used in a large-scale epidemiological study [36]. The questionnaire consists of three sections: activity at home, work and recreation. Permission was obtained from Wareham and Jakes to adapt and use the instrument for this study.

The content of the EPAQ-2 was assessed for cultural appropriateness by a panel that included experts from inside and outside the Health Promotion Board (Singapore). Minor modifications were made to the list of recreation activities. Activities that were not relevant to local context, such as “digging, shovelling or chopping wood” were removed, and replaced with common local activities not included in the version developed for use in Europe, such as Tai Chi. Seventeen women at a workplace (not involved in the study) assessed the ease of reading using the Flesch reading ease score. They also provided feedback on the ease of understanding and the ease of completing the modified EPAQ-2. Minor changes were made to the language of instructions on the questionnaires to further increase ease of understanding. Prompts were added in sections where extra information needed to be provided, for example duration of each session recreation activity, to facilitate thorough completion of the questionnaire.

A copy of the EPAQ-2 was sent to each participant through the workplace coordinator. The participants completed the questionnaire independently and submitted it to the investigator at each data collection point. The investigator checked that the EPAQ-2 was completed according to instructions.

### Self-efficacy data collection

Self-efficacy was measured using the osteoporosis self-efficacy scale developed and evaluated by Horan et al. in 1998 [37]. Written permission was sought from the authors to use the instrument.

The content of the questionnaire was assessed for appropriateness to local context by a panel that included experts internal and external to the Health Promotion Board (Singapore). It was also validated for internal consistency and test-retest repeatability through an evaluation process involving 17 women at a workplace (not involved in the study). The original content was found to be relevant to the local context and the questionnaire to have appropriate reliability for use in this study.

Each subject was sent a physical copy of the questionnaire through the workplace coordinator. The completed questionnaires were returned to the workplace coordinator who collated the submissions and dispatched them to the investigator.

### Socio-demographic information and other measures

Demographic and other health information was collected using questionnaires. At baseline, this included smoking and alcohol habits, family history of osteoporosis, indicators of socio-economic status (such as personal and household income, education level), religious preference (potentially relevant to diet and physical activity), marital status and number of children in the household.

### Intervention methodologies

Subjects from workplaces assigned to the intervention group received three intensive workshops targeting behaviour change. The intervention design had a strong focus on behavioural strategies and was participatory in nature. Bandura’s Self–Efficacy Model was used to guide the workshop design for the intervention group. Bandura’s model states that self-efficacy affects health behaviour and its determinants by influencing goals and aspirations. The stronger the perceived self-efficacy, the higher the goals people set for themselves and the firmer their commitment to them [38]. Bandura also proposed that individuals with high efficacy view impediments as surmountable by improvement of self-management skills, that they persevere and stay the course in the face of difficulties [38].

Guided by these principles, the workshop design focused on individual goal setting and on building skill sets to attain individual goals. The design avoided presentation style communication and focused on behavioural strategies such as participatory skill building through hands-on activities, goal setting exercises, peer support and problem solving discussions. Attention was placed on helping participants identify individual barriers and build their capacity to overcome them. The intervention also addressed diet and physical activity as different entities that required different behavioural strategies. Though guided by the same principles, the workshops for diet and physically activity were unique in the nature and design of their activities.

### Intervention strategy development for calcium intake

Our study reviewed the content of interventions that reported positive outcomes for dietary calcium intake. These studies described strong behavioural strategies using participatory activities such as food preparation and tasting, nutrition label reading exercises, group discussions with exchange of ideas [14,15,39]. Their interventions also placed emphasis on using specific examples relevant to the participants' lifestyles and tastes [14,15,40], as well incorporating local food sources into activities. Evidence also suggested that the provision of calcium intake feedback might be an effective tool to improve the behaviour [39]. These elements were used to guide the development of intervention content in this study.

In addition, we planned to incorporate quantitative and qualitative dietary information collected at baseline to help tailor intervention strategies. The dietary records would be inspected to identify the common food sources of calcium amongst the study population, as well as their consumption patterns and volumes. This information would also be used to tailor strategies that would be relevant to individual participants.

Common barriers to consuming calcium rich foods, identified in previous research include the perception that these foods are higher in price, are mainly dairy products and are high in fat. Taste aversion, mainly to dairy, has also been highlighted as a barrier. These issues were addressed individually in different components of the workshop. Food tasting was a very effective strategy to expose subjects to a wide range of foods that can provide a substantial calcium boost. The ability to correctly read and interpret nutrition labels was identified as a necessary skill to facilitate selection of calcium rich foods. Practical sessions on reading of food labels were included in the intervention.

Subjects in both the intervention and control group received individual feedback on their average calcium intake based on the diet record they submitted. Although the subjects in the control group received similar information about their calcium consumption, the intervention group had the benefit of using this individualised information during the workshop to develop a strategy to attain their recommended daily allowance whilst the former did not. A logic model of the intervention can be found in Figure 3.

### Intervention strategy development for physical activity

Local media campaigns and community based health promotion activities on osteoporosis prevention have had a stronger focus on diet compared to physical activity in the lead up to the time of the study. Limited local public education sources were dedicated to discussing the impact of physical activity on bone health and more importantly, the types of physical activity that can reduce the risk of osteoporosis.

Physical activity behaviour can have very different psychosocial mediators from dietary behaviour. In this study, physical activity was regarded as a unique behaviour that required a different set of intervention strategies to that for dietary calcium intake.

The types of physical activity that can affect bone mineral density, risk of osteoporosis and risk of fractures are described as load bearing and resistance training exercises [41,42]. The intensity of activity is also critical. Evidence indicates that only moderate to vigorous level of load-bearing physical activity can affect bone density in important sites such as the hips, a vulnerable site for osteoporotic fractures [43,44].

Studies about physical activity behaviour in the context of bone health are limited to adolescent populations and were carried out mainly in school settings. Strategies for adolescents cannot be used to guide the content development for this study. There are many studies on prescriptive exercise regimes but the interventions were aimed at studying bone density in response to exercise interventions, not physical activity uptake behaviour. The attrition rates for these studies are very high as behaviour modifications were not a focus.

In the absence of evidence in adult populations, this study evaluated the methodologies from studies that targeted general physical activity in community settings, including workplaces. It is important to note that whilst this study used workplaces as settings or delivery platforms, it did not have sufficient resources to implement changes to workplace environments and policies. This study aimed to improve individuals' self-efficacy to bring about behaviour change. Workplaces provided a platform to support change at the individual level through the provision of infrastructure for communication, peer support and common interest.

Evidence indicates behavioural strategies to be superior to cognitive strategies [19,45]. Meta-analyses of physical activity interventions emphasise the importance of behavioural interventions, which include goal setting, self-monitoring, physical activity behaviour feedback, consequences, exercise prescription and cues [19,45]. Our study adhered to these recommendations when designing intervention activities. In addition, emphasis was placed on providing opportunities to sample a variety of the targeted physical activities. These included take home activity samplers in many formats, including DVDs.

The first workshop discussed the relationship between load bearing and resistance training exercises on bone cell formation and bone modelling. This served to communicate the key message that specific types of exercise are needed to protect and promote bone health. The third workshop was focused entirely on helping participants identify their barriers to increasing their physical activity level and to help them overcome the barriers with individually tailored strategies.

The intervention for physical activity was facilitated by a physiotherapist and the investigator. The aims of the intervention were to encourage participants to problem solve and develop strategies to help them increase their moderate to vigorous intensity load-bearing physical activity by 60 minutes per week. Each participant would record their weekday and weekend routine to be shared within a group. In the design of the intervention, peer support was identified as an important mediator for behaviour change and hence the emphasis on group work. This allowed participants to identify common barriers to change, develop strategies together and support one another through problem solving on common issues. Workshop participants shared a common work environment and would be able to develop workplace based strategies based on shared experience. This facilitated discussion as participants shared many ideas that were based on common experience, such as workplace stair access for opportunistic physical activity, discussion of suitable walking routes around the workplaces and sharing of information about exercise facilities near the work premises.

Almost 50% of Singapore adults cited lack of time as a barrier to leisure time physical activity [22]. Unique strategies were developed for this study to facilitate the attainment of the physical activity goal with minimal disruption to the participants’ routine. One important strategy was to introduce short bouts of exercise breaks (5–10 minutes) during television viewing time or work time, which many participants would regard as achievable and sustainable. Participants would devise different types of 5–10 minute exercise routines that required minimal room and could be carried out easily at home or at the workstation. Resources, such as an exercise CD and a 10-minute exercise poster with instructions and illustrations would be provided to each participant. The latter was developed specifically for this intervention.

### Control arm

Participants in control/standard care workplaces would receive a resource kit with general print resources on bone health and osteoporosis prevention. They would received a report with their average calcium intake based on their dietary records but would not be provided with recommendations for change.

### Proposed data analysis

All analyses will follow intention-to-treat principles when comparing intervention and control arms [46]. Data from the second follow up will be analysed to compare short-term changes after the intervention. Data from the third follow up will be analysed to assess sustainability of any observed changes. The primary dependent variables are calcium intake (milligrams per day), moderate to vigorous intensity load-bearing physical activity level (duration in minutes per week).

### Cluster level analysis that adjusts for individual covariates and baseline measures

The main hypotheses will be tested using cluster-level analyses. This approach adheres to the recommendation of the 2004 Consort Statement for cluster randomised trials to fully account for the clustering effect [46,47] and is recommended for studies with small number of clusters [47,48]. Individual-level analysis using multi-level/mixed models was considered, but this study does not have the required cluster numbers for multilevel modelling as recommended by some analysts (minimum recommended is 15 cluster per arm) [47].

This study will use the two-stage adjusted analysis based on cluster summaries developed by Hayes and Moulten (2009, pp182-184) [47]. In stage one, SPSS linear regression will be run to generate an unstandardised residual for each subject (the difference between the observed value and the fitted value from the model for each individual participant). Baseline calcium value for each subject will be controlled for in the linear regression model. Other variables that were identified as potential confounders will also be included in the model as factors or covariates. This step incorporates repeated measures into the analysis and generates the covariate-adjusted residuals for each individual.

Cluster summaries for the covariate-adjusted residuals are then generated using SPSS descriptive statistics. These summaries are for stage two of the analysis, which is the cluster level analysis. The cluster means of the residuals are then compared in a cluster level analysis using a weighted *t*-test, with weighting based on cluster size (number of participants per cluster).

### Process to outcome analysis

Analysis will also test for the mediating effects of self-efficacy (SE) scores on calcium intake and physical activity measures. The relationships between the SE scores and the outcome measures will be examined at baseline and each follow up by exploring if changes in the self-efficacy scores are predictive of changes in the outcome measures in this cohort. The intervention theory and design assumes self-efficacy to be a strong mediator of outcome and this assumption will be tested and discussed.

## Discussion

This study design was developed in response to the need for a well-designed population-based intervention to target osteoporosis prevention behaviours. Although dietary and physical activity behaviour interventions have been extensively researched, limited investigation has been carried out to study the specific domains of these two behaviours relevant to bone health and osteoporosis prevention.

### Strengths

To our knowledge, this was the first population-based osteoporosis prevention intervention that has attempted to intervene with diet and physical activity components as unique health behaviours, and the first that adopts a self-efficacy-based theoretical model to guide behavioural strategies for both health behaviours. The study was also unique in the selection of workplaces as a vehicle for the delivery of the interventions. While worksites have been a popular platform for health behaviour-directed disease prevention, none to date has focused on osteoporosis prevention. Worksites are also ideal setting for identifying higher risk population such as individuals with sedentary occupations.

The participatory components in the study were expected to strengthen the intervention efficacy. The study utilised baseline assessment both to tailor the interventions as well as for evaluation purposes.

The intervention treated calcium intake and physical activity as individual health behaviours. This is vital as the two behaviours have their unique characteristics and barriers and need to be addressed with very different strategies. The study was able to implement separate workshops for calcium intake and physical activity thus facilitating the development of unique strategies for each of the behaviours.

### Limitations

The outcomes measures in this study were all derived from self-reports, which may incur potential bias. Steps were taken in this study to ensure the self-report measurement tools in this study were valid and reliable for the study population. Nevertheless, the osteoporosis preventive potential of the intervention would be more accurately and precisely assessed using objective bone mass density or other biomarker measures. However, resource and feasibility limitations excluded these possibilities. This is an important consideration for future studies in this area.

This study utilised the social structure and existing infrastructure within workplaces to deliver the interventions, but it did not have the capacity to influence workplace policies and practices to address sedentarism at the organisational level. This is a limitation in the study and an important consideration for future research, as intervention strategies to reduce workplace sitting time are rapidly developing.

At the time of the study, published ICC values for the targeted outcome and mediators were either limited or absent. In the absence of published ICCs, we adopted a conservative approach of assuming large ICCs in the calculations. This might have inflated the required number of clusters. However, it is also possible that the study will be underpowered to account for potential confounding. We will publish the observed ICC values for calcium intake, physical activity measures and self-efficacy scores in the reporting of the main results of the study. A retrospective calculation of the number of clusters and power based on the actual observed ICCs will be reported to compare with the original estimates.

High attrition rate is often a problem in population based intervention studies. A higher dropout rate is anticipated in this study because of loss of subjects through work factors such resignation and corporate restructuring. In an attempt to maintain cluster sizes, there was deliberate over-recruitment of within cluster subjects by 30 percent. However, some degree of selection bias may occur if attrition is high.

## Abbreviations

ICC: Intraclass correlation coefficient; MET: Metabolic equivalent of task; MET-hour: Metabolic equivalent of task per hour; RDA: Recommended daily allowance

## Competing interests

The authors declare that they have no competing interests.

## Authors’ contributions

AMT designed the study and the intervention and drafted the manuscript. AD. LM contributed to the drafting and revision of the manuscript. PH contributed to the design of the study and the intervention program, and revised the manuscript. RS contributed to the dietary evaluation component of the study design. All authors read and approved the final version of the manuscript.

## Pre-publication history

The pre-publication history for this paper can be accessed here:

http://www.biomedcentral.com/1471-2458/13/405/prepub
